# Dietary Factors Associated with Plasma Thyroid Peroxidase and Thyroglobulin Antibodies

**DOI:** 10.3390/nu9111186

**Published:** 2017-10-28

**Authors:** Antonela Matana, Vesela Torlak, Dubravka Brdar, Marijana Popović, Bernarda Lozić, Maja Barbalić, Vesna Boraska Perica, Ante Punda, Ozren Polašek, Caroline Hayward, Tatijana Zemunik

**Affiliations:** 1 Department of Medical Biology, School of Medicine Split, University of Split, Šoltanska 2, 21000 Split, Croatia; antonela.boljat@mefst.hr (A.M.); mpopovic@mefst.hr (M.P.); mbarbali@mefst.hr (M.B.); vesna.boraska@mefst.hr (V.B.P.); 2 Department of Nuclear Medicine, University Hospital Split, Spinciceva 1, 21000 Split, Croatia; veselakbsplit@yahoo.com (V.T.); d.brdar.dr@gmail.com (D.B.); ante.punda@mefst.hr (A.P.); 3 Department of Pediatrics, University Hospital Split, Spinciceva 1, 21000 Split, Croatia; blozic@kbsplit.hr; 4 Department of Public Health, School of Medicine Split, University of Split, Šoltanska 2 , 21000 Split, Croatia; opolasek@gmail.com; 5 MRC Human Genetics Unit, University of Edinburgh, Western General Hospital, Crewe Road, Edinburgh EH4 2XU, UK; caroline.hayward@igmm.ed.ac.uk

**Keywords:** autoimmune thyroid diseases, thyroid peroxidase antibodies, thyroglobulin antibodies, dietary habits

## Abstract

The knowledge about dietary habits and their influence in the development of autoimmune thyroid disease is insufficient. The aim of this study was to analyse the association of dietary factors and plasma thyroid peroxidase antibodies (TPO-Ab) and/or thyroglobulin antibodies (Tg-Ab). The study enrolled 1887 participants originating from the South Croatia. Participants with elevated plasma TPO-Ab and/or Tg-Ab were defined as cases (*n* = 462) and those with TPO-Ab and/or Tg-Ab within referent values were defined as controls (*n* = 1425). Dietary intake was evaluated according to a food frequency questionnaire containing 58 food items. Principal component analysis was used to group food items into dietary groups. We used logistic regression analysis to examine dietary groups associated with positive plasma TPO-Ab and/or Tg-Ab. The results indicate that the dietary group with frequent consumption of animal fats and butter is associated with positive plasma TPO-Ab and/or Tg-Ab (*p* = 0.01). The dietary group with frequent consumption of vegetables as well as the dietary group with high consumption of dried fruit, nuts, and muesli are associated with negative findings of TPO-Ab and/or Tg-Ab (*p* = 0.048 and *p* = 0.02, respectively). We showed that the anti-inflammatory dietary groups are associated with the negative findings of plasma TPO-Ab and/or Tg-Ab.

## 1. Introduction

Thyroid disorders are, beside diabetes mellitus, the most frequent disorders affecting endocrine systems. Autoimmune thyroid diseases (AITD) such as Hashimoto disease and Graves disease are characterised by an autoimmune reaction against thyroid autoantigens [1]. One of the first findings of AITD is positive serum thyroid peroxidase antibody (TPO-Ab) [2]. Genetic background significantly contributes to the development of autoimmune thyroid diseases (70% to 80%), but their occurrences are also associated with different environmental factors (20% to 30%) [1].

It has been shown that increased occurrence of thyroid autoantibodies is the result of iodine sufficiency or excessive iodine intake [3,4,5].

Among other environmental factors, smoking was associated with increased risk for Graves disease (GD) but not with Hashimoto thyroiditis (HT), while moderate alcohol consumption had a protective role in the development of GD and HT [1,3]. Low selenium intake and low serum vitamin D levels were shown to be associated with higher risk of AITD, but the data is still inconclusive [1,3]. Stress could be a risk factor for GD, but has not been studied enough for HT [1,3]. A lower incidence of GD was linked to estrogen intake [1]. Infection with Yersinia enterocolitica was shown to be associated with GD, while enterovirus infection was associated with HT [1,3]. Molecular mimicry is a possible mechanism for pathogen association with infections and immune response. Recent studies based on bioinformatics data support the triggering role of several bacterial and viruses in the onset of AITD [6]. The intake of different drugs was related with GD and HT [1,3].

However, there are no comprehensive studies investigating dietary habits and their influence on AITD to date. Only two studies analysed the prevalence of self-reported hypothyroidism and hyperthyroidism in relation to dietary habits, and found a protective effect of vegan diet from hypothyroidism, and both vegan and vegetarian diets from hyperthyroidism [7,8].

In the current study, we evaluated the association of dietary factors and plasma thyroid antibodies. The goal of the study was to identify groups of food items that are associated with positive or negative findings of plasma TPO-Ab and/or Tg-Ab.

## 2. Materials and Methods

This case-control study was performed using the data collected through the “10,001 Dalmatians” project [9,10]. The project provided comprehensive data about the dietary habits of participants, as well as stored plasma samples that were used for biochemical measurements in this study. We included 1887 participants originating from the Dalmatian region of South Croatia (921 from the island of Korcula and 966 from the city of Split). The participants were adult volunteers from the general population (over 18 years of age). A written informed consent was obtained from participants and the study protocol was approved by the Ethical board of the University of Split, School of Medicine (No: 2181-198-03-04-14-0031).

Participants were allocated in either group based on thyroid antibodies status; subjects were considered as cases if their level of TPO-Ab and/or TgAb were higher than referent values (TPO-Ab > 16 IU/mL and Tg-Ab > 100 IU/mL), while those with negative findings (within referent values) of TPO-Ab and Tg-Ab were determined as controls. Controls who were taking thyroid medication (*n* = 27) or who had undergone thyroid surgery (*n* = 10) were excluded from the analysis. Finally, a total of 462 cases and 1388 controls were included in the study. Out of 462 cases, five had other autoimmune diseases: four had psoriasis, and one had psoriatic arthritis. In the control group, one out of 1388 participants had systemic lupus erythematosus.

Plasma TPO-Ab and Tg-Ab were determined by the sandwich chemiluminescence immunoassay method in the Laboratory of Biochemistry, Department of Nuclear Medicine, University Hospital Split (Split, Croatia). The immunoassay was conducted in a fully automated instrument “Liaison” Biomedica Chemiluminescence Analyser (DiaSorin, Saluggia, Italy) using in vitro assays for the quantitative determination of TPO-Ab and Tg-Ab in the plasma.

Dietary intake was assessed with a food frequency questionnaire (FFQ) that consisted of 54 foods and beverages. The frequency of food intake was measured using five categories: every day, 2–3 times a week, once a week, occasionally, and never. Additionally, there were four questions regarding the frequency of fat consumption with three possible answers (always, sometimes, never). For analysis, frequency categories for each food item were converted into an equivalent weekly intake as follows: every day (converted to 7 times a week), 2–3 times a week (2.5), once a week (1), occasionally (0.5—once in two weeks), and never (0). Responses on the frequency of fat consumption were also converted to an equivalent weekly intake as follows: always (7 times a week), sometimes (2.5 times a week), and never (0 times a week).

The data are presented as the mean ± standard deviation (SD) for continuous variables and as frequencies (percentages) for categorical variables in Table 1. The χ^2^ test was used to assess the differences between groups for categorical variables, and the *t*-test was used for numerical variables. Principal Component Analysis (PCA) was used to identify underlying patterns of food consumption to reduce the list of 58 food items to key dietary groups (factors), such that the foods in each dietary group tend to be consumed equally often. The factors were rotated by orthogonal transformation (varimax rotation) to obtain a more interpretable structure. Propriety of using factor analysis was tested by the Kaiser-Meyer-Olkin measure of sampling adequacy and Bartlett’s test of sphericity. Factors with an eigenvalue greater than 1.0 were retained. A food item was considered to load on a given factor if the absolute factor loading value was >0.3 for that factor and ≤0.3 for all other factors. The factor loading of a food item increases as the contribution to the corresponding dietary group increases. Each factor contains a distinct set of food items. Factor-specific scores were calculated using the regression method and assigned to each participant. Odds ratio (OR) and 95% confidence intervals (CI) were calculated by multiple logistic regression to examine factors associated with positive plasma TPO-Ab and/or Tg-Ab, i.e., between cases and controls. The logistic regression model included gender and dietary factors. *p*-values of less than 0.05 were considered as statistically significant. Statistical analysis was conducted using Statistical Package Software for Social Science, version 16 (SPSS Inc., Chicago, IL, USA).

## 3. Results

A total of 462 cases and 1388 healthy individuals were enrolled in this study. The characteristics of study participants are shown in Table 1. A significantly higher number of women had positive TPO-Ab and/or Tg-Ab, while there was no statistically significant difference in age and body mass index (BMI) between cases and controls (Table 1).

Suitability of the respondent data for factor analysis was supported by the Kaiser-Meyer-Olkin measure of sampling adequacy (0.77) and Bartlett’s test of sphericity (*p* < 0.001). Factor analysis revealed 19 dietary factors, which explained 54.76% of the total variance in food intake. The factors were generally in accordance with conventional dietary groups. Loading values for factors are presented in Table 2.

**Figure 1:**
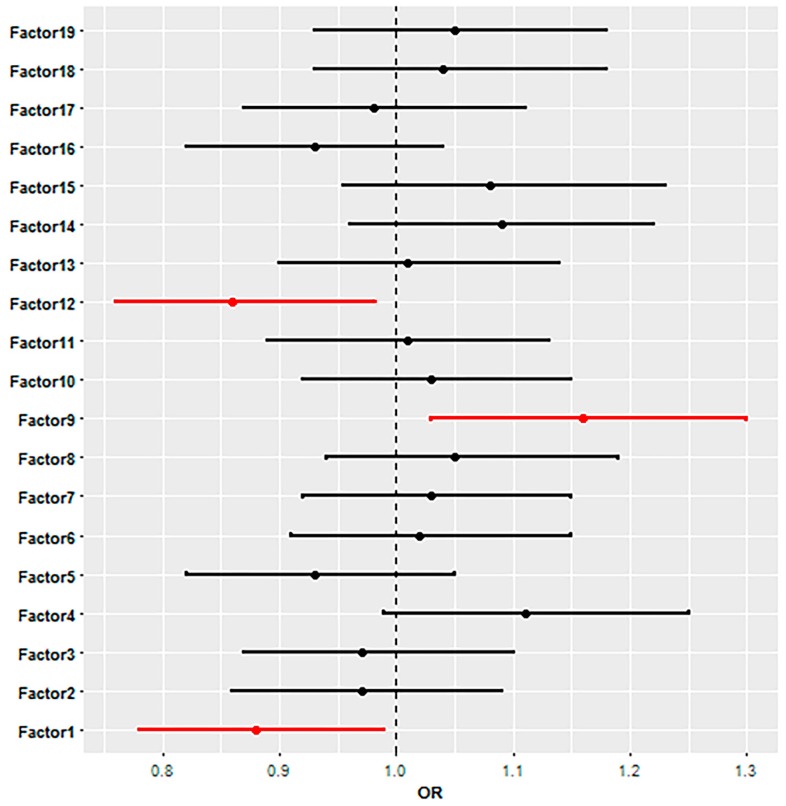
Odds ratios (OR) and 95% confidence intervals obtained from the logistic regression analysis for the association of dietary groups (factors) with plasma TPO-Ab and/or Tg-Ab. Significant dietary groups are coloured in red, while not-significant dietary groups are coloured in black. If the OR and the lower limit of the 95% confidence interval are above 1, the dietary group is positively associated with plasma TPO-Ab and/or Tg-Ab, whereas if the OR and the upper limit of the 95% confidence interval are below 1, the dietary group is negatively associated with plasma TPO-Ab and/or Tg-Ab.

Logistic regression analysis revealed that the dietary group with high loadings for root vegetables, flower vegetables, leafy vegetables, fruity vegetables, and legumes was negatively associated with the plasma TPO-Ab and/or Tg-Ab (OR = 0.88, 95% CI 0.78–0.99, *p* = 0.048). The dietary group with high loadings for dried fruit, nuts, and muesli was also negatively associated (OR = 0.86, 95% CI 0.76–0.98, *p* = 0.02), while the dietary group with high loadings for butter and animal fats was positively associated with plasma TPO-Ab and/or Tg-Ab (OR = 1.16, 95% CI 1.03–1.30, *p* = 0.01). Other dietary groups showed no association with plasma TPO-Ab and/or Tg-Ab. Results from the logistic regression analysis are shown in Figure 1.

## 4. Discussion

This study has shown that the dietary group (factor 9) with frequent consumption of animal fats and butter was associated with positive plasma TPO-Ab and/or Tg-Ab, while the dietary groups (factors 1 and 12) with frequent consumption of different sorts of vegetables, dried fruit, nuts, and muesli were associated with negative findings of TPO-Ab and/or Tg-Ab.

Furthermore, the study showed that a higher number of women had positive TPO-Ab and/or Tg-Ab, which is in accordance with previously published studies [1,3,11]. Age and body mass index did not show association with positive TPO-Ab and/or Tg-Ab. The influence of body mass index is controversial in the literature, while the prevalence of positive thyroid antibodies increases with age [11,12,13].

Butter and animal fats are rich in saturated fatty acids (SFA) [14,15]. Vegetables, nuts, and cereals are rich in polyunsaturated fatty acids (PUFA) [16]. All the food items from dietary groups 1 and 12 of this study are rich in *n*-6 and *n*-3 PUFAs. Dietary *n*-6 and *n*-3 PUFAs are imbedded into the cell plasma membranes, where they serve as precursors in the synthesis of eicosanoids. These two types of PUFAs have opposite effects on inflammatory responses, whereas *n*-6 PUFAs serve as the precursors of inflammatory eicosanoids while *n*-3 generates anti-inflammatory eicosanoids [17]. The rate of *n*-6 conversion into *n*-3 in humans is below 5% and, consequently, the levels of anti-inflammatory eicosanoids are almost completely linked to the amount of dietary *n*-3 consumption [14]. Eicosanoids such as eicosapentaenoic acid (EPA) and docosahexaenoic acid (DHA) are involved in the regulation of pro-inflammatory cytokines and act as anti-inflammatory mediators, and therefore have benefits in autoimmune diseases such as rheumatoid arthritis [17].

Studies performed in experimental animals showed that a diet enriched with *n*-3 fatty acids suppresses the inflammation that accompanies autoimmune reactions [18,19,20,21]. The results indicate that *n*-3 PUFAs reduce the differentiation of Th17 cells from naive CD4+ T cells by modifying the lipid rafts regions in their plasma membrane and decreasing the formation of IL-6 receptors [18]. It is known that Th17 cells positively correlate with serum TPO-Ab and Tg-Ab [22]. Recently published studies showed that Th17 cells play the main role in the pathogenesis of AITD [23,24].

Food items included in dietary groups 1 and 12 are rich in *n*-3 fatty acids and seem to suppress the production of plasma TPO-Ab and Tg-Ab. On the contrary, saturated fatty acids of animal origin (dietary group 9) seem to have harmful influence on TPO-Ab and/or Tg-Ab production. Frequent consumption of animal fats and butter could result in a low ratio of *n*-3 fatty acids (anti-inflammatory)/*n*-6 fatty acids (inflammatory) in the diet and thus a lack of suppression of Th17 T cells differentiation, resulting in the promote TPO-Ab and/or Tg-Ab production [18,22].

Phytosterols are present in moderate and small amounts in nuts and vegetables, respectively, and have shown immunomodulatory and anti-inflammatory properties [25,26]. They are contained in dietary groups 1 and 12, and are associated with negative findings of plasma TPO-Ab and/or Tg-Ab. One of the proposed ways of their immunomodulation activity is the reduction of IL-6 plasma levels [25,26]. Since IL-6 is the main stimulator in Th17 cells differentiation, the reduction of IL-6 could be the reason behind the protective effect of phytosterols in the pathogenesis of AITD.

Polyphenols, abundant micronutrients in the diet, are components of fruits and vegetables. They are known for their anti-inflammatory, immunomodulatory, and antioxidative effect in the body [27,28]. Gallic acid could have a major role in the explanation of the beneficial effect of dried fruit and muesli (dietary group 12) on positive plasma TPO-Ab and/or Tg-Ab observed in this study. Gallic acid is a component of red fruit, but it can also be found in apple peels and grapes [28]. Red fruits are often consumed as dried fruit, and some of them are common components of muesli. Kuppan et al. found that treatment of human monocytes with gallic acid reduces the expression of IL-6 gene [29]. Hence, the reduction of IL-6 plasma levels could have a suppression effect in differentiation of Th17 cells involved in the pathogenesis of AITD.

To date, two studies have been published regarding diet and hypothyroidism/hyperthyroidism. The authors reported an association of vegan diet and lower risk of hypothyroidism [7]. The same group of authors also showed that vegan and vegetarian diets are associated with lower risk of hyperthyroidism [8]. The authors suggest a possible protective effect of polyphenols (such as flavonoids) against autoimmune processes, but a detailed explanation on the molecular level is not provided. They also discuss the possible effect of environmental toxins from food on the microbiome and their possible stimulation of autoimmune disease [7,8].

Benvenga et al. showed an association of lower serum thyroid autoantibodies and oily fish consumption and hypothesised about the protective effect of *n*-3 fatty acids [30]. In our study, dietary group 2, composed of different seafoods, did not reach significance in the association. The possible reason for this could be the presence of squids, octopus, shells, crabs, and white fish in that group, which are not as rich in *n*-3 fatty acids as blue (oily) fish [31]. Milerova et al. showed a positive correlation of phytoestrogen genistein with TgAb level in the sera of school children screened for iodine deficiency. However, relatively low iodine intake might be related with this finding [32].

Saturated fatty acids of animal origin, mostly presented in pro-inflammatory Western diets, showed a negative effect, while anti-inflammatory vegetarian and especially vegan diets showed a beneficial effect in the pathogenesis of other autoimmune diseases such as rheumatoid arthritis, multiple sclerosis, and systemic lupus erythematosus [33,34,35,36].

The present study has several limitations. This is a cross-sectional study and therefore only associations, and not causations, were inferred. Furthermore, all variables regarding food consumption were self-reported. Food frequency questionnaire had limitations for quantitative assessment of food intake; however, it provided relevant dietary information. Although we controlled for sociodemographic variables, unmeasured confounding factors could be present, including inflammatory parameters that may influence associations.

## 5. Conclusions

In summary, the present study demonstrated the association of animal fats and butter consumption with the positive plasma TPO-Ab and/or Tg-Ab. Vegetables, dried fruit, nuts, and muesli consumption was associated with the negative findings of TPO-Ab and/or Tg-Ab. In light of recently published studies, we discussed the possible protective effect of *n*-3 fatty acids from vegetables and nuts on the IL-6 receptors of the CD4+ T cells plasma membranes, and the suppression of their differentiation in Th17 T cells involved in the pathogenesis of AITD. Likewise, we discussed the suppressive effect of phytosterols and polyphenols found in vegetables, nuts, dried fruit, and muesli on IL-6 secretion. It seems that the frequent consumption of anti-inflammatory food items (vegetables, nuts, dried fruit, and muesli, as presented in dietary groups 1 and 12) is associated with the negative findings of plasma TPO-Ab and/or Tg-Ab.

## Figures and Tables

**Table 1:** Differences between cases and controls in sociodemographic characteristics.

Variable	Cases	Controls	*p* Value
Gender			<0.001 ^a^
Males	119 (25.8%)	584 (42%)	
Females	343 (74.2%)	804 (58%)	
Age (year)	53.66 ± 13.65	52.72 ± 14.93	0.45 ^b^
BMI	27.78 ± 7.29	27.95 ± 7.79	0.67 ^b^

BMI: Body mass index. ^a^ χ^2^ test, ^b^ *t*-test.

**Table 2:** List of food items and factor loadings for 19 dietary groups (factors) identified using principal component analysis.

Factors	Food Items (Factor Loadings)
Factor 1	Root vegetables (0.79), flower vegetables (0.76), leafy vegetables (0.74), fruity vegetables (0.70), legumes (0.54)
Factor 2	Squid and octopus (0.78), sea-food (shells, crab) (0.71), blue fish (0.67), dried fish and salted sardines (0.56), white fish (0.49)
Factor 3	Chocolate (0.78), cookies (0.76), cakes (0.69), bonbons (0.51)
Factor 4	Salami (0.69), canned meat derivates (0.62), sausages (0.57), eggs (0.45), bacon (0.36)
Factor 5	Cedevita (powder based vitamin juice) (0.68), fruits juices (0.62), refreshing non-alcoholic drinks (0.60)
Factor 6	Bran bread (−0.81), white bread (0.73)
Factor 7	Full-fat cheese (0.69), cottage cheese (0.63), hard cheese (0.52), sour cream (0.46)
Factor 8	Venison (0.76), fish derivates (0.53)
Factor 9	Butter (0.73), animal fats (0.70)
Factor 10	Internal organs (0.73), lamb (0.52), pork (0.37)
Factor 11	Mushrooms (0.64), canned and pickled vegetables (0.61), potato (−0.30)
Factor 12	Muesli (0.70), dried fruit (0.56), nuts (0.44)
Factor 13	Hard liquor (0.68), vegetables juices (0.57), powder soups (0.33)
Factor 14	Tea (0.64), olive oil (0.40)
Factor 15	Milk (0.70), coffee (0.51), yoghurt (0.49), fresh fruits (0.40)
Factor 16	Chicken (0.68), turkey (0.68)
Factor 17	Beef (0.73), veal (0.34)
Factor 18	Macaroni or rice (0.63), jam and marmalade (0.37), fruit compote (0.33)
Factor 19	Plant oil (0.77)

Absolute values ≤ 0.3 were excluded for simplicity.
